# Voluntary Running in Young Adult Mice Reduces Anxiety-Like Behavior and Increases the Accumulation of Bioactive Lipids in the Cerebral Cortex

**DOI:** 10.1371/journal.pone.0081459

**Published:** 2013-12-11

**Authors:** Iván J. Santos-Soto, Nataliya Chorna, Néstor M. Carballeira, José G. Vélez-Bartolomei, Ana T. Méndez-Merced, Anatoliy P. Chornyy, Sandra Peña de Ortiz

**Affiliations:** 1 Department of Biology, University of Puerto Rico, Rio Piedras Campus, San Juan, Puerto Rico; 2 Metabolomics Research Center, University of Puerto Rico, Rio Piedras Campus, San Juan, Puerto Rico; 3 Department of Chemistry, University of Puerto Rico, Rio Piedras Campus, San Juan, Puerto Rico; 4 School of Sciences and Technology, Universidad del Este, Carolina, Puerto Rico; 5 High Performance Computing Facility, University of Puerto Rico, Central Administration, San Juan, Puerto Rico; University of Victoria, Canada

## Abstract

Combinatorial therapies using voluntary exercise and diet supplementation with polyunsaturated fatty acids have synergistic effects benefiting brain function and behavior. Here, we assessed the effects of voluntary exercise on anxiety-like behavior and on total FA accumulation within three brain regions: cortex, hippocampus, and cerebellum of running versus sedentary young adult male C57/BL6J mice. The running group was subjected to one month of voluntary exercise in their home cages, while the sedentary group was kept in their home cages without access to a running wheel. Elevated plus maze (EPM), several behavioral postures and two risk assessment behaviors (RABs) were then measured in both animal groups followed immediately by blood samplings for assessment of corticosterone levels. Brains were then dissected for non-targeted lipidomic analysis of selected brain regions using gas chromatography coupled to mass spectrometry (GC/MS). Results showed that mice in the running group, when examined in the EPM, displayed significantly lower anxiety-like behavior, higher exploratory and risky behaviors, compared to sedentary mice. Notably, we found no differences in blood corticosterone levels between the two groups, suggesting that the different EPM and RAB behaviors were not related to reduced physiological stress in the running mice. Lipidomics analysis revealed a region-specific cortical decrease of the saturated FA: palmitate (C16:0) and a concomitant increase of polyunsaturated FA, arachidonic acid (AA, omega 6-C20: 4) and docosahexaenoic acid (DHA, omega 3-C22: 6), in running mice compared to sedentary controls. Finally, we found that running mice, as opposed to sedentary animals, showed significantly enhanced cortical expression of phospholipase A2 (PLA_2_) protein, a signaling molecule required in the production of both AA and DHA. In summary, our data support the anxiolytic effects of exercise and provide insights into the molecular processes modulated by exercise that may lead to its beneficial effects on mood.

## Introduction

Anxiety disorders comprise the most common group of mental illnesses in the United States [1]–[3]. Recently, life style changes such as exercise have been proposed as possible complimentary modalities to manage this growing health problem [4]. In fact, several studies have demonstrated that voluntary exercise has beneficial effects on spatial learning and memory assessed in the Morris water maze, which has inherent stress and anxiety components [5]–[10]. Similar findings were obtained when comparing exercise versus sedentarines using the fear conditioning, a learned anxiety paradigm [11]–[13].

The Elevated Plus maze (EPM) has classically been used to assess innate, non-learned, anxiety like behavior in rodents [14], [15]. In addition to measuring anxiety-like behavior by comparing the time animals spent in open versus closed areas of the maze, the EPM allows the monitoring of defensive, protective, and vigilant behaviors [12]. Previous studies utilizing the EPM and an Open Field test observed reduced anxiety in mice after voluntary wheel running [16]–[18]. In contrast, other studies reported increased anxious behavior [19], [20] and corticosterone derivates in feces [19], contradicting several studies that support an anxiolytic effect of exercise [16]–[18]. Hence, the effects of voluntary exercise on anxiety-like behavior are still controversial.

Recently, several researchers combined voluntary exercise with dietary supplementation using omega-3 docosahexaenoic (DHA) and omega-6 arachidonic (AA) fatty acids (FA). DHA supplementation potentiated the known beneficial effects of exercise on enhanced spatial learning [6]–[10], and increased expression of various molecules involved in synaptic plasticity [21], [22]. DHA and AA are polyunsaturated FA mostly acquired exogenously through diet or produced endogenously from linoleic and α-linoleic FA, which are acquired from the diet [23]–[25]. Polyunsaturated FA have been reported to be important in neural development, cell signaling, and membrane fluidity [23]–[25], which are roles that might be important for the effects of exercise in the brain including neurogenesis [26]–[28], as well as the induction of neurotrophins and molecules involved in synaptic plasticity [29]–[35].

Here, we hypothesized that voluntary exercise in young adult mice reduces anxiety-like behavior and modulates the content of FA in discrete brain regions. Overall, our results support previous findings reporting that voluntary exercise reduces anxiety-like behavior, while at the same time increasing certain types of risk-taking behaviors. Moreover, our studies identified biochemical differences in the brains of running and sedentary mice that could be related to reduced anxiety-like behavior between the groups examined. Specifically, voluntary wheel running induced cortex-specific elevation of DHA and AA with corresponding reduction of palmitate (PA) contents. These behavioral and biochemical findings may help to unravel the mechanisms by which voluntary exercise modulates anxiety-like behavior and may eventually help to determine how life style changes influence mental health.

## Results

### Voluntary wheel use increases throughout the four weeks of training

**Figure 1:**
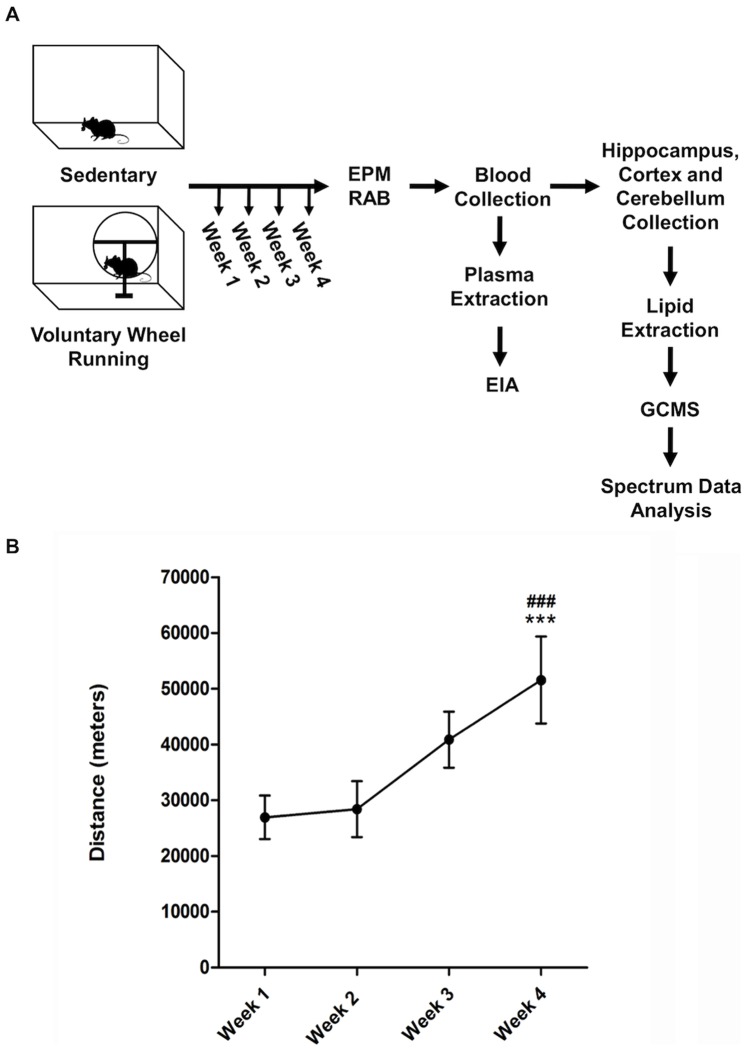
Experimental Design and Distance Run As a Measure of Voluntary Exercise. **A**. Schematic representation of the voluntary wheel running protocol design, including additional behavioral and molecular procedures. After four weeks of running wheel training, mice were evaluated with the EPM/RAB measures. Next, blood was collected, brains were extracted, and brain regions dissected. Later, plasma samples were obtained from blood to examine corticosterone levels with an Enzyme Immunoassay (EIA). FA were extracted from the dissected brain regions and non-targeted neurolipidomic analysis was performed using GC/MS. **B**. Distance in meters ran by the mice in the Running group (N = 18). One-Way repeated measures ANOVA identified a significant increase in the distance ran throughout the weeks of training (F (3, 71)  = 7.489, ***P<0.0005). Bonferroni post-testing showed a specific significant increase in running distance when comparing week 1 versus week 4 and week 2 versus week 4 (***P<0.001 each comparison). Data are expressed as the mean ± SEM.

**Figure 1A** depicts our initial experiments designed for determining the effects of 30 days of voluntary running on anxiety-like behavior. As studied by others [16]–[18], while sedentary mice were placed in individual cages, running mice had free access to a running wheel having an automatic counter. **Figure 1B** depicts the average distance ran by the animals for each of the four weeks of our study. As weeks progressed, mice with access to the wheel showed a significant increase in running distance as measured by meters ran per week. Repeated measures one-way analysis of variance (ANOVA) identified a significant increase in the meters ran through the weeks of training (F (3, 71)  = 7.489, ***P<0.0005). Bonferroni post-testing showed a specific significant increase in running distance when comparing week 1 versus week 4 and week 2 versus week 4 (***P<0.001 each comparison).

### Mice subjected to voluntary wheel running have a reduction of anxiety-like behavior and an increase in risk taking behavior

**Figure 2:**
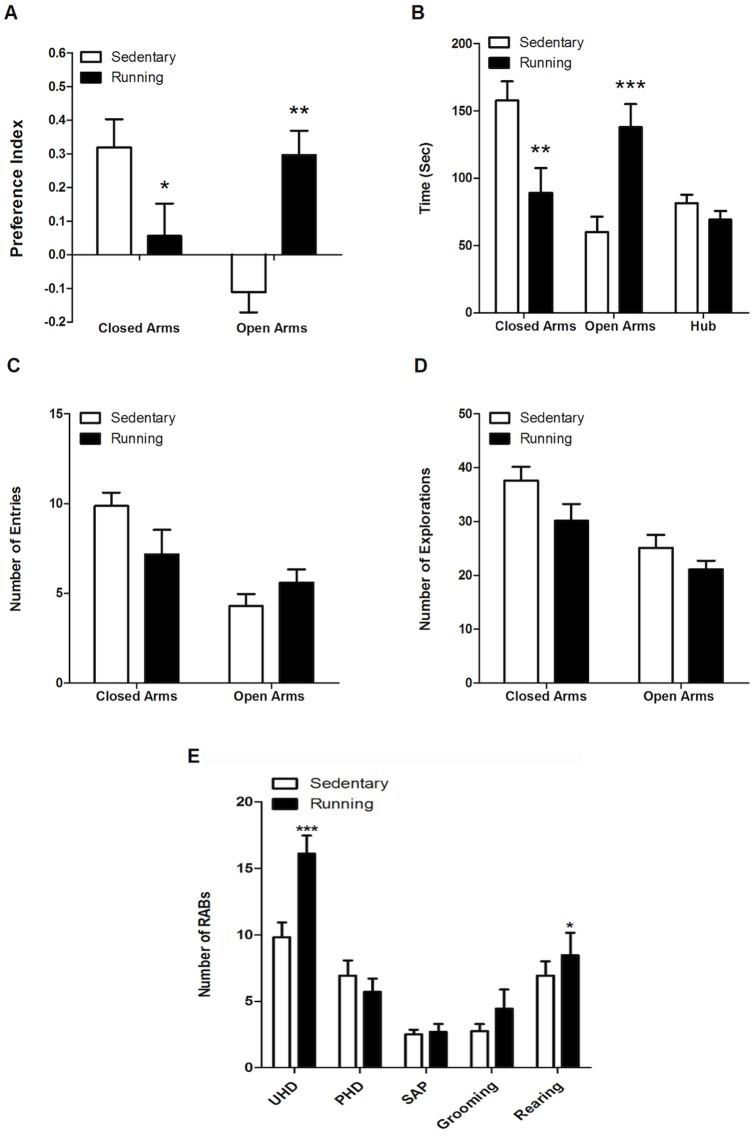
Behavioral Results in the Elevated Plus Maze. **A**. Bar graph depicting the preferences of running (black) and sedentary (white) mice for closed versus open arms in the EPM. The results show that running mice displayed a significant preference for the open arms (**P<0.01). **B**. Bar graph depicting the time spent by running and sedentary mice on open versus closed arms. Post-testing identified that running mice spent significantly more time in the open arms (***P<0.001) and less time on the closed arms (**P<0.01) compared to sedentary mice. **C**. The number of entries to the open and closed arms of the maze by running and sedentary mice. Both sedentary and running mice displayed more entries to the closed arms than to the open arms of the EPM (Two-Way ANOVA, Running Factor: F (1, 64)  = 0.59, P = 0.4470; Arms Factor: F (1, 64)  = 15.13, ***P<0.0005; Interaction: F (1, 64)  = 4.70, *P<0.05). The significant interaction between the running and arms factors indicates that animals from both groups displayed an opposite preference for entering the respective arms, as seen in the graph. **D**. Bar graph depicting the number of explorations of closed and open arms by the running and sedentary mice. Running affected significantly the type of arms explored (Two-Way ANOVA: Running: (F (1, 64)  = 5.30, P>0.0246); Arms (F (1, 64)  = 18.57, ***P<0.0001; Interaction; (F (1, 64)  = 0.49, P>0.4886). **E**. Bar graph depicting the RABs examined. Unprotected and protected head dippings (UHD and PHD, respectively), stretch attendance postures (SAPs), grooming, and rearing. General Linear Model (GLM) MANOVA revealed an overall significant effect by exercise (Exercise: F (5, 33)  = 5.4619, **P<0.01). Multiple post-testing confirmed that running animals had significantly higher number of UHD (F (1, 33)  = 15.9054, ***P<0.0005) and Rearing (F (1, 33)  = 5.553, *P<0.05), but no significant difference in PHD (F (1, 33)  = 3.5576, P>0.05), SAPs (F (1, 33)  = 1.9902, P>0.1), or grooming (F (1, 33)  = 0.3035, P>0.5) were found.

After four weeks of voluntary exercise, mice were subjected to the EPM test with concomitant measurements of Risk Assessment Behaviors (RAB). We first wanted to determine whether sedentary versus running mice displayed different preferences for spending time in the closed versus the open arms of the EPM. Arm preference indices were calculated by first summating the time spent in either the two closed or the two open arms and subtracting this value from the time spent in the hub. The resulting value was divided by the summation of the time spent in open arms plus the time spent in the closed arms. These analyses began to unveil important differences between sedentary and running mice in terms of their preferences for closed versus open arms in the EPM. After we performed the analysis, we found a significant interaction between the exercise and arm factors (Two-Way ANOVA, Running Factor: F (1, 64)  = 0.84; P = 0.3614; Arms Factor: F (1, 64)  = 1.43, P = 0.2365; Interaction: F (1, 64)  = 17.94, ***P<0.0001). Bonferroni post-testing identified specific significant differences between sedentary and running mice. As seen in **Fig. 2A** **,** running mice displayed a significant preference for the open arms (**P<0.01) and significantly diverted from the closed arm (*P<0.05), compared to sedentary mice. In agreement with these findings, as seen in **Fig. 2B** we also identified a significant effect in the time the mice spent in the areas of the maze. We found a significant interaction between the exercise factor and the maze areas factor in relation to the time spent on open versus closed arms, and the time spent in the hub (Two-Way ANOVA, Running Factor: F (1, 96)  = 0.01, P = 0.9332; Maze Areas Factor: F (2, 96)  = 6.60, **P>0.005; Interaction: F (2, 96)  = 15.55, ***P<0.0001). Bonferroni post-testing identified specific significant differences between sedentary and running mice in terms of the time each group spent within closed (**P<0.01) versus open (***P<0.001) arms, respectively. In contrast, there was no difference between sedentary and running mice in regards to the time they spent in the hub area of the EPM (P>0.05). No statistically significant differences between running and sedentary animals was observed with respect to the number of entries to either closed or open arms in the EPM (**Fig. 2C**; Two-Way ANOVA, Running Factor: F (1, 64)  = 0.59, P = 0.4470; Arms Factor: F (1, 64)  = 15.13, ***P<0.0005; Interaction: F (1, 64)  = 4.70, *P<0.05). The number of entries represents the number of times each animal entered its complete body into an arm followed by a complete retraction. The plotted data in **Fig. 2C** shows a reduced number of entries to the closed arms by running animals, compared to sedentary ones, while at the same time showing higher numbers of entries by running mice into the open arms, compared to sedentary mice. Thus, the significant statistical interaction between the Running and Arms factors in the data analyzed in **Fig. 2C**, supports the conclusion that voluntary exercise, compared to sedentariness, reduces the entries into closed arms, while simultaneously increasing the entries to open arms in the EPM.

Finally, as seen in **Fig. 2D**, running affected significantly the type of arms explored, be it open or closed, yet not the number of explorations within them (Two-Way ANOVA: Running: (F (1, 64)  = 5.30, *P<0.05); Arms (F (1, 64)  = 18.57, ***P<0.0001); Interaction; (F (1, 64)  = 0.49, P = 0.4886). Since explorations represent the number of times animals introduce their heads and frontal paws within an arm followed by a complete retraction, these findings potentially reflect the fact that running animals were less likely to shy away from open arms than sedentary mice, an interpretation that is in agreement with our data in **Fig. 2B**. The EPM data show that running mice compared to sedentary controls: i) have significantly higher preference for spending time in open versus closed arms (**Fig. 2A**); ii) spend significantly more time exploring open versus closed arms (**Fig. 2B**); iii) have a tendency to display lower numbers of body entries into closed arms, as opposed to open arms, while simultaneously displaying a tendency for higher number of entries into the open arms (**Fig. 2C**); and finally iv) show reduced numbers of explorations followed by complete retractions toward open arms, but not the closed arms (**Fig. 2D**). In summary, the behavioral differences between running and sedentary mice in our EPM studies suggest that voluntary exercise reduces anxiety-like behavior, since running mice displayed a higher willingness to be environmentally exposed on the open arms. On the other hand, running mice were more likely to reject spending their time secluded in the closed arms, a behavior that was preferred by their sedentary counterparts.

We also monitored the frequency of several behavioral postures, within those a couple of RABs, while the animals where exploring the EPM. The RABs examined included unprotected and protected head dippings (UHD and PHD, respectively), stretch attendance postures (SAPs), as well as grooming and rearing (**Fig. 2E**). UHD, PHD. SAP, grooming and rearing data obtained from sedentary and running animals were analyzed using General Linear Model (GLM) MANOVA. The GLM-MANOVA effects summary revealed an overall significant effect by exercise (Exercise: F (5, 33)  = 5.4619, **P<0.01). In addition, GLM-MANOVA identified significant differences between sedentary and running animals with respect to UHD (F (1, 33)  = 15.9054, ***P<0.0005) and Rearing (F (1, 33)  = 5.553, *P<0.05). No difference between groups with respect to PHD (F (1, 33)  = 3.5576, P>0.05), SAPs (F (1, 33)  = 1.9902, P>0.1), or grooming (F (1, 33)  = 0.3035, P>0.5) was observed. UHD behavior is as an exploratory movement in which the animal's head is protruding over the side of the open arms and down towards the floor, that is, the animal is taking the risk, while on the open arms of the EPM, of extending its head downward to observe and explore below. During rearing, an exploratory behavior engaged while animals are moving around their environment attempting to contact relevant stimuli, mice put their weight on the hind legs, raise the forelimbs from the ground, and extend the head upwards. Increased rearing is indicative of increase explorative behavior and curiosity, resulting from decreased anxiety and an increased sense of safety. The fact that mice in the running group displayed increased UHD and rearing behaviors, compared to sedentary controls, also suggest that voluntary running reduces anxiety and increases the sense of safety that is accompanied during the engaging in risk-taking behaviors.

### Sedentary and running mice had no differences in plasma corticosterone levels

Plasma was extracted from sedentary and running mice that had experienced four weeks of voluntary wheel running and were then tested in the EPM. No significant differences in corticosterone levels (pg/ml) were identified between Sedentary and Running mice (Student's t-test: Sedentary, 1445±253.2 vs. Running 1394±200.8; t_(29)_  = 0.1584, P = 0.8752, N = 16, per group). The fact that there were no significant differences in corticosterone levels between either set of sedentary and running animals, even though running animals displayed significantly lower levels of anxiety-like behavior according to the EPM/RAB measurements (**Figure 2**), suggested to us that this exercise-related effect on mood could be associated to biochemical or molecular changes in the brain not directly related to corticosterone.

### Non-targeted neurolipidomics revealed specific differences in FA abundances in the cortex of running versus sedentary young adult mice

Following EPM testing and blood collection for corticosterone analysis (see above), brain tissue was used for lipid extraction. We performed gas chromatography/mass spectrometry (GC/MS) analyses to profile the content of FA methyl esters in the three different brain regions studied in running and sedentary mice: cerebellum, cortex, and hippocampus. Our studies detected 16 FA methyl esters regardless of tissue localization or exercise activity. Out of these, six were saturated FA (Myristic (C14:0), Palmitic (C16:0), Stearic (C18:0), Arachidic (C20:0), Behenic (C22:0), and Lignoceric acid (C24:0), five were monounsaturated FA (Palmitoleic (C16:1), Oleic (C18:1, omega-9), Eicosenoic (C20:1), Erucic (C22:1), and Nervonic acid (C24:1); and five were polyunsaturated FA (Eicosadienoic (C20:2), Homo-g-linoleic (C20:3, omega-6), Arachidonic (AA, C20:4, omega-6), Adrenic (C22:4, omega-6), and DHA (C22:6, omega-3). **Table 1** presents the different percents of each individual FA from the total of all FA in different brain regions of both running and sedentary mice.

**Table 1:** FA content in the brain regions of sedentary and running young male C57BL/6J mice.

Name	FA	CX SED	CX RUN	H SED	H RUN	CER SED	CER RUN
**Myristic Acid**	**C14:0**	0.26±0.11	0.21±0.05	1.24±1.06	0.20±0.08	0.34±0.08	0.33±0.08
**Palmitic Acid**	**C16:0**	39.34±4.74	**27.40±1.53**	31.70±2.88	30.50±2.54	24.00±2.30	25.12±1.82
**Stearic Acid**	**C18:0**	25.60±1.74	23.40±1.03	27.18±1.67	26.94±2.05	11.740±5.00	11.67±5.00
**Arachidic Acid**	**C20:0**	2.04±1.63	0.75±0.36	0.03±0.02	0.49±0.21	1.20±0.27	1.37±0.58
**Behenic Acid**	**C22:0**	0.17±0.04	0.34±0.19	0.10±0.03	0.22±0.08	0.40±0.14	0.79±0.36
**Lignoceric Acid**	**C24:0**	0.33±0.24	0.32±0.13	0.09±0.04	0.39±0.23	0.98±0.39	0.87±0.34
**Palmitoleic Acid**	**C16:1**	1.09±0.29	1.07±0.27	0.39±0.13	0.43±0.32	1.08±0.27	1.13±0.20
**Oleic Acid ω −9**	**C18:1**	15.32±3.04	13.38±2.70	18.27±1.22	18.34±1.35	20.93±2.26	17.93±2.61
**Eicosenoic Acid ω −9**	**C20:1**	0.54±0.23	1.17±0.45	1.01±0.27	2.15±0.87	5.41±0.66	4.73±0.88
**Erucic Acid ω −9**	**C22:1**	0.16±0.08	0.20±0.08	0.06±0.02	0.14±0.06	0.17±0.03	0.33±0.14
**Nervonic Acid ω −9**	**C24:1**	0.08±0.04	0.16±0.07	0.20±0.14	0.25±0.15	0.98±0.68	1.14±0.71
**Eicosadienoic Acid**	**C20:2**	0.86±0.40	0.51±0.13	ND	0.21±0.11	0.94±0.62	1.18±0.61
**Homo-g-Linoleic Acid ω −6**	**C20:3**	6.11±2.68	0.82±0.18	ND	0.66±0.26	0.89±0.24	1.74±1.33
**Arachidonic Acid ω −6**	**C20:4**	0.92±0.67	**11.32±0.72**	6.28±1.09	8.47±1.38	8.22±0.95	8.20±1.68
**Adrenic Acid ω −6**	**C22:4**	3.03±1.83	3.83±1.86	6.06±1.44	2.58±1.02	2.72±0.66	4.36±0.97
**Docosahexaenoic Acid ω −3**	**C22:6**	0.78±0.53	**9.98±2.13**	6.42±1.60	7.35±1.14	8.07±1.16	5.49±2.47

Data are expressed as the percent of the total methylated FA ± SEM, N = 6–8. FA designated with (ND) were not detected in the sample. FA  =  Fatty Acids, CX SED  =  Cortex Sedentary, CX RUN  =  Cortex Running, H SED  =  Hippocampus Sedentary, H RUN  =  Hippocampus Running, CEB SED  =  Cerebellum Sedentary, and CEB RUN  =  Cerebellum Running. Shown in bold are the values representing the significant increases in running versus sedentary animals for Palmitic Acid, Arachidonic Acid ω −6, and Docosahexaenoic Acid ω −3, respectively.

**Figure 3:**
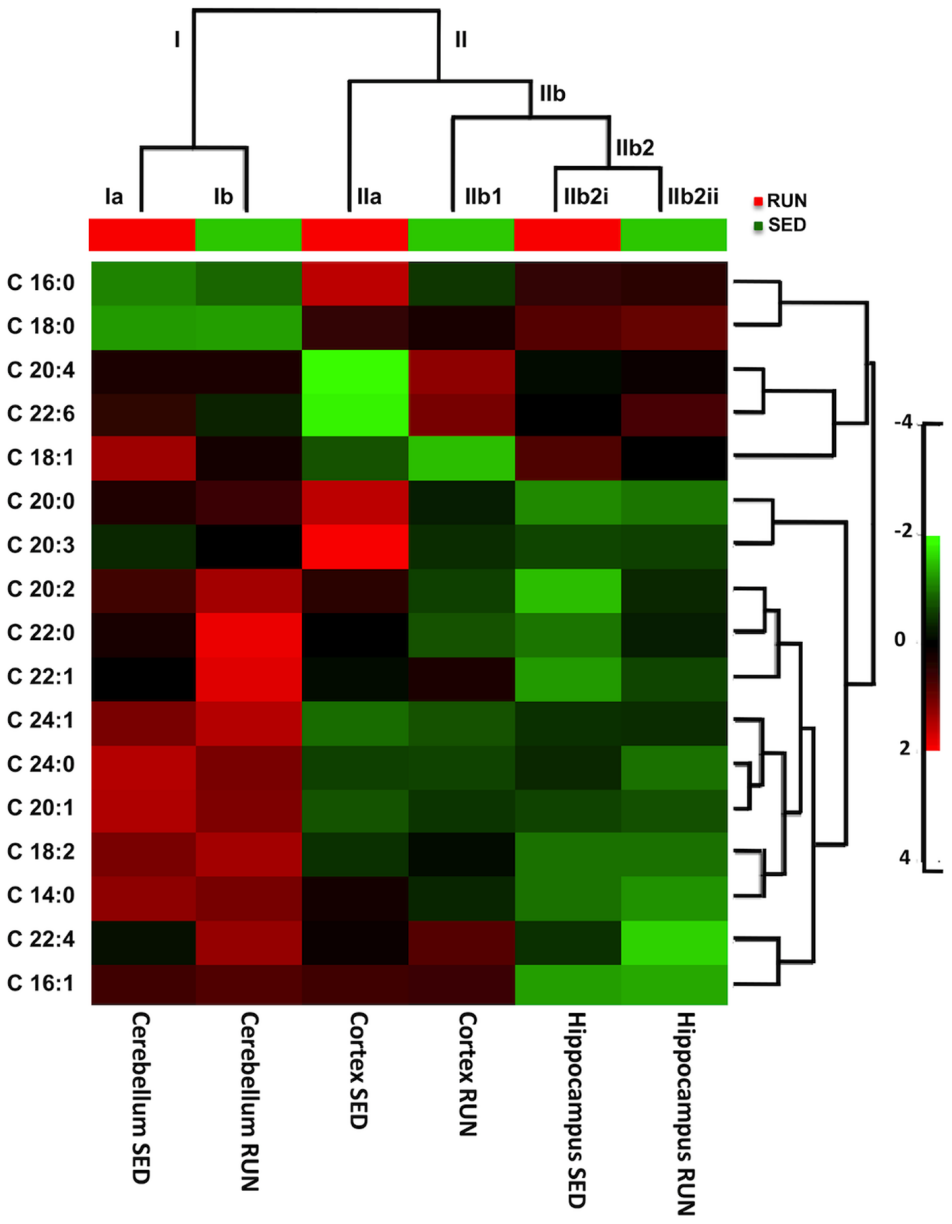
Hierarchical Cluster Analysis of Neurolipidomic Data Obtained by GC/MS. Raw data was subjected to analysis at the MetaboAnalyst website. A Hierarchical Clustering and Heat map were generated with the data utilizing logarithmic transformation and a column wise normalization. The program produced two superior clusters: one superior cluster separates the cerebellum of sedentary mice from all other regions and the other superior cluster created a subordinate cluster that clearly separates the cortex from the hippocampus of both groups of animals and the cerebellum of running mice. This subordinate cluster of the cortex also makes a separation between running and sedentary mice. The heatmap revealed a significant increase in the abundance of AA and DHA in the cortex of running mice compared to the cortex of sedentary mice.

We used hierarchical clustering [36] as a bioinformatics tool to gain initial insights from our GC/MS data. **Figure 3** depicts the resulting dendrogram with its two superior clusters (Clusters I and II) separating the FA profile of the cerebellum (Cluster I), from that of cortex and hippocampus (Cluster II) of both running and sedentary mice, respectively. Cerebellar Cluster I, generated subordinate clusters Ia and Ib, separating sedentary and running groups, respectively. Cluster II branched into subordinate clusters (IIa and IIb), revealing a distinct separation of the FA profile of the cortex of sedentary mice from that of running animals, the latter also sub-clustering with the hippocampus data for both groups of animals. Sub-cluster IIa, which included FA from the cortex of sedentary mice, separated from sub-cluster IIb1, which in turn included FA from the cortex of running mice. Hippocampal FA from running mice clustered together with the hippocampal FA from sedentary mice (subordinated IIb2 cluster), suggesting that similar to the cerebellum (Cluster I), there were no differences in hippocampal FA abundance between both groups. Overall, the results supported the notion of a possible difference between the nature and abundance of FA of sedentary versus running mice in the cortex, but not the cerebellum or the hippocampus.

**Figure 4:**
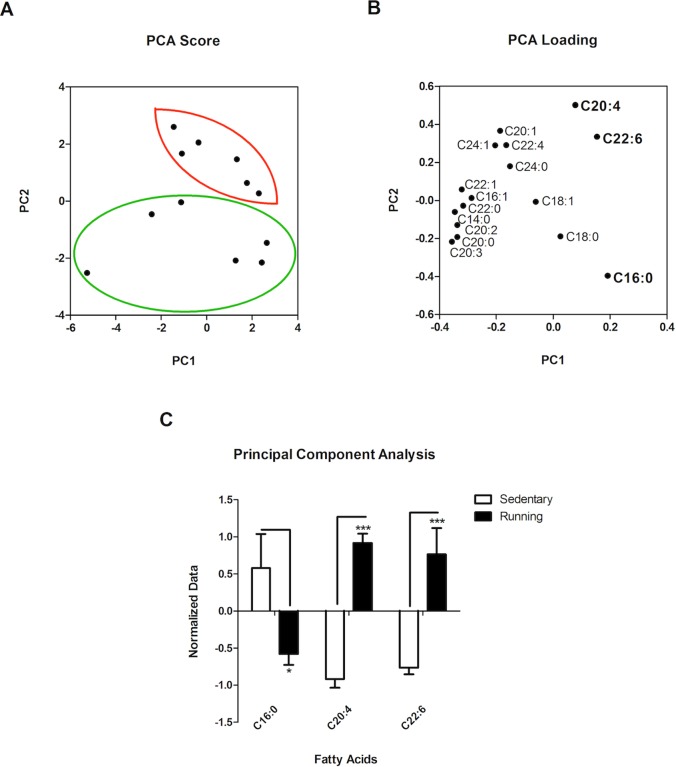
PCA Analysis of GC/MS Data. The cortical nerolipidome raw data normalization at MetaboAnalyst was subjected to PCA variance analysis. **A**. Variance of the cortical samples showing a clear difference between samples belonging to Running mice (dots within red oval) and their Sedentary counterparts (dots within green oval). **B**. Variance between the different FA detected in the cortical samples from running and sedentary mice. AA and DHA had the most significant variance showing a significant increase in running mice, compared to sedentary controls. PA also had a significant variance showing decreased levels in running mice. **C**. Bar graph depicting the differences in PA (*P<0.05), AA (***P<0.001), and DHA (***P<0.001) normalized abundance in sedentary and control mice. Data are expressed as the mean ± SEM.

Principal component analysis (PCA) of the cortical data obtained from both groups supported the notion that there are cortical differences in FA between sedentary and running mice. **Figure 4** depicts PC1, showing the highest variations between groups (**Fig. 4A**). The most discriminatory FA were identified by inspection of PC1 and PC2 loadings (**Fig. 4B**). **Figure 4C** depicts the normalized data from the PCA analysis for FA with the highest significant variance: palmitic acid, PA (C16:0), arachidonic acid, AA (C20:4), and decohexahenoic acid, DHA (C22:6). Two-Way ANOVA revealed that voluntary exercise had a significant effect in the abundance of specific FA species (Two-Way ANOVA: FA Abundance Factor, F (2, 30)  = 0.000, P = 1.000; Exercise Factor, F (1, 30)  = 12.29, **P<0.005; Interaction F (2, 30)  = 20.51, ***P<0.0001). Bonferroni post-tests revealed a significant decrease in cortical PA (*P<0.05) and a concomitant significant increase of cortical AA (***P<0.001) and DHA (***P<0.001) in response to exercise. Overall, the data suggests that cortical FA profiles, specifically of PA, AA, and DHA, of running versus sedentary mice share the highest distance separation indicating different FA abundances, compared to those observed in the other clusters and comparisons examined.

### Analysis of GC/MS data revealed a decrease of PA and an increase of AA and DHA in the cortex of young adult running mice

**Figure 5:**
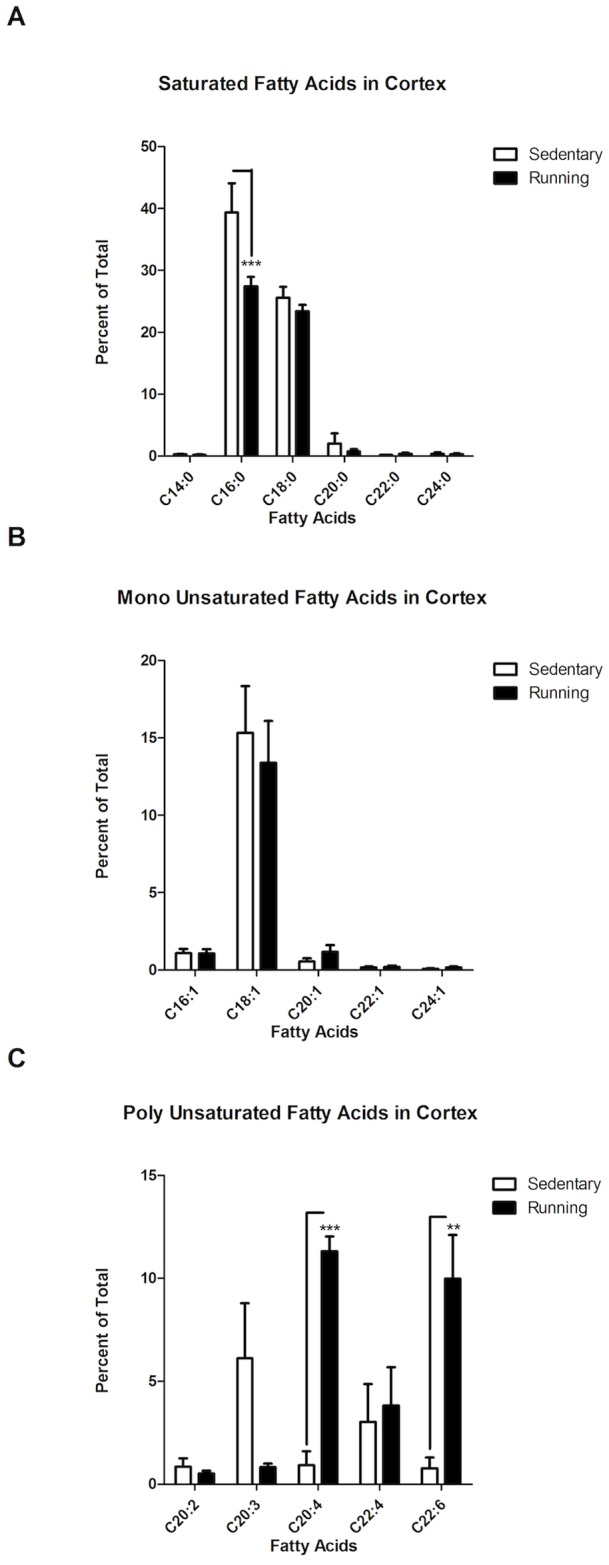
Cortical Lipid Profiling of Running and Sedentary Mice. Percent of total was calculated for each of the 16 FA. FA from the cortex of running and sedentary mice were separated for this analysis into Saturated, Monounsaturated and Polyunsaturated FA. **A**. Saturated FA: Two-Way ANOVA identified a significant difference with respect to the abundance across the different FA species (***P<0.0001) and also with respect to exercise (**P<0.01). Bonferroni post-testing revealed a specific statistically significant and dramatic reduction in PA cortical levels in running versus sedentary mice (***P<0.001). **B**. Monounsaturated FA: Two-Way ANOVA identified a significant difference with respect to the abundance across the different FA species (***P<0.0001), as oleic acid displayed a significantly higher abundance than the rest of the monounsaturated FA. No difference in monounsaturated FA abundance was detected in response to exercise (P = 0.7733). **C**. Polyunsaturated FA: Results from Two-Way ANOVA identified significant effects by exercise on Polyunsaturated FA abundance across the different FA species (**P<0.01). Bonferroni post-testing analysis revealed a significant increase of AA (***P<0.001) and DHA (**P =  <0.01), caused by running. Data are expressed as the mean ± SEM.

We next focused on our brain regional neurolipidomic data to carry out an in depth analysis to identify the most relevant specific differences in FA abundances between sedentary and running mice (**Fig. 5**). We first analyzed our hippocampal and cerebellar data, which did not display any potential differences in FA abundances between sedentary and running mice, in the hierarchical clustering analysis (**Fig. 3**). While statistical analyses unveiled a significant difference in the abundances among the various saturated FA species analyzed within these regions, no significant effects of exercise were identified. Statistical analyses of the cerebellar data yielded the following results: saturated FA (Two Way ANOVA: FA Species Factor, F (5, 60)  = 39.12, ***P<0.0001); monounsaturated FA (Two Way ANOVA: FA Species Factor: F (4, 50)  = 91.56, ***P<0.0001); polyunsaturated FA (Two Way ANOVA: FA Species Factor, F (5, 60)  = 10.73, ***P<0.0001). Similarly, hippocampus statistical analyses yielded the following results: saturated FA (Two Way ANOVA: FA Species Factor, F (5, 60)  = 231.5, ***P<0.0001); monounsaturated FA (Two Way ANOVA: FA Species Factor, F (4, 50)  = 292.4, ***P<0.0001); polyunsaturated FA (Two Way ANOVA: FA Species Factor, F (5, 60)  = 27.19, ***P<0.0001). Overall, for both the cerebellum and the hippocampus, the significant differences in FA abundance were related only to the biochemical nature of FA species profiled, rather than to the experience of exercise or sedentariness.

In contrast to the results described above for the hippocampus and cerebellum, and in agreement with our findings depicted in **Figs. 3** **–** **4**, analysis of our cortical lipidomic data showed significant and specific effects by exercise for distinct FA. As seen in **Fig. 5A**, running and sedentary mice displayed a statistically significant difference in the abundance of saturated FA (Two-Way ANOVA: FA Species Factor, F (5, 60)  = 168.3, ***P<0.0001; Exercise Factor, F (1, 60)  = 7.382, **P<0.01). The analysis also identified a significant interaction between exercise and FA abundance (F (5, 60)  = 4.146, **P<0.001), suggesting that the observed differences between groups might be associated to specific FA. Indeed, Bonferroni post-testing analysis revealed a significant cortical decrease in PA levels as a result of running (***P<0.001). Other saturated FA, such as Myristic (C14:0), Stearic (C18:0), Arachidic (C20:0), Behenic (C22:0), and Lignoceric acid (C24:0) showed similar abundance in both of sedentary and running mice (P>0.05).

As was the case with the cerebellar and hippocampal lipidomic profiles (see above), cortical monounsaturated FA (**Fig. 5B**) differed significantly in abundance with respect to each other, but not due to exercise (Two-Way ANOVA: FA Species Factor: F (4, 50)  = 45.16, ***P<0.0001; Exercise Factor, F (1, 50)  = 0.08385, P = 0.7733). The abundance of oleic acid in the cortex was significantly higher than that of the other monounsaturated FA profiled in our study, irrespective of Exercise. No significant interaction was identified between the factors analyzed (F (4, 50)  = 0.2866, P = 0.8853). Bonferroni post-testing did not identify any specific significant difference in the abundance of monounsaturated FA in the cortex of sedentary versus running mice (P>0.05).

On the other hand, analysis of polyunsaturated cortical FA unveiled important and significant differences among sedentary and running groups (**Fig. 5C**). Two-Way ANOVA identified a significant effect of exercise on polyunsaturated FA levels (Exercise Factor: F (1, 60)  = 7.559, **P<0.01), although no significant effect was identified in relation to the group of polyunsaturated FA analyzed (FA Species Factor: F (5, 60)  = 2.299, P>0.05). Importantly, however, a significant interaction between Exercise and FA Species (F (5, 60)  = 5.704, ***P<0.001) was determined, suggesting that exercise did cause a significant and specific effect on particular polyunsaturated FA. Bonferroni post-testing confirmed such assumption by revealing a specific significant increase of AA (***P<0.001) and DHA (**P<0.01) abundances in the cortex of running, compared to sedentary, mice. Other polyunsaturated FA remained unchanged in both groups.

### Voluntary exercise significantly increased levels of PLA_2_ in the cortex of running mice

**Figure 6:**
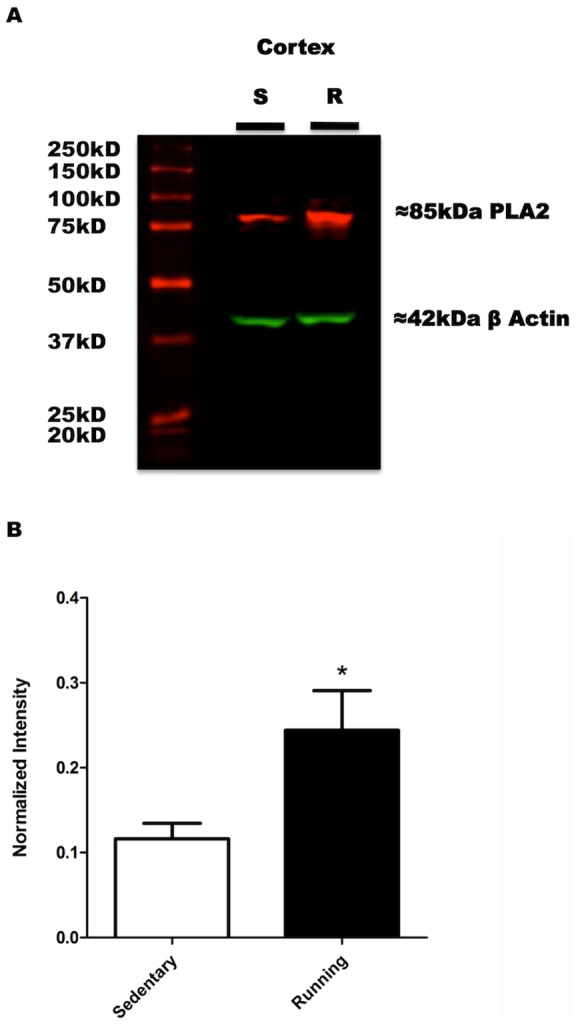
Western Blot of Cortical PLA_2_. Proteins were extracted from separate groups of running and sedentary mice than those used for behavioral and lipidomic analyses. Western blots for PLA_2_ immuno-detection were performed using cortical protein samples extracted from running and sedentary mice. **A**. Representative Western Blot image obtained from the Odyssey Infrared Imaging System. The molecular weight marker is shown in lane 1, followed by cortical protein extract samples from sedentary (lane 2) and running (lane 3) mice. The 85 kDA IRD680 dyed red band represents PLA_2_, while the 42 kDA IRD800 dyed green band represents β Actin. As seen in the representative Western blot, cortical PLA_2_ was expressed at higher levels in running versus sedentary mice. **B**. Fluorescent in tensities of PLA_2_ labelings were normalized against those of β-Actin in order to analyze the expression data. As seen in the bar graph, results revealed a significant elevation of non-phosphorylated total PLA_2_ levels in running mice, compared to sedentary controls (*P<0.05). Data are expressed as the mean ± SEM.

Because of the increase in AA and DHA levels in the cortex in running animals versus sedentary controls (**Figs. 3** **–** **5**), we set out to determine if phospholipase A2 (PLA_2_) expression levels were also elevated in the cortex as a result of running. Different subtypes of PLA_2_ are known to be required for intracellular increases of both AA and DHA. PLA_2_ cleaves acyl ester bonds at the sn-2 position of membrane phospholipids producing free FA [37]–[38]. Cortical protein extracts were obtained from mice after four weeks of voluntary wheel running and Western blot immuno-detection for PLA_2_ was performed (**Fig. 6A**). Western blot analysis of cortical extracts revealed a significant elevation of non-phosphorylated total PLA_2_ levels in the cortex in running versus sedentary mice (**Fig. 6A, B**), as determined by unpaired Student's t-test analysis (Student's t-test: Sedentary, 0.1164±0.01812 vs. Running 0.2442±0.04667; t_(10)_  = 2.552, *P<0.05, N = 6). The fact that running increases cortical PLA_2_ concomitantly with AA and DHA, suggests that such polyunsaturated FA changes could be associated to the activation of intracellular signaling cascades by voluntary physical exercise involving the PLA_2_ enzyme.

## Discussion

### Anxiolytic Effects of Voluntary Wheel Running in Young Adult Mice

Here we show that voluntary wheel running decreases anxiety-like behavior in young adult mice. Specifically, running increased EPM behavioral endpoints representing fearless exploratory and risk-taking conduct, demonstrated by the unprotected head dipping and rearing, suggesting that exercised animals were able to maintain a stronger sense of safety compared to sedentary controls. By utilizing the EPM as a classic model to study unlearned anxiety behavior, these results expand other studies investigating the effects of exercise on anxiety-like behavior [16]–[20], [36]. We only focused on the EPM for several reasons. The EPM is one of the most popular screening methods to measure innate (unlearned) anxiety and anxiety-like-behaviors and it is simple and easy to use [16]–[18]. On the other hand, the fear-conditioning task was not used to evaluate anxiety here in order to avoid confounding factors involving learning and memory associated to such task, processes both known to be affected by exercise [37], [38]. In addition, as in all the studies examining the effects of exercise on animals, sedentary controls were caged individually since this type of study is required to ensure that the effects of exercise are measured in individual animals. However, housing mice under isolation conditions may pose negative neurodevelopmental effects [39], [40]. Thus, the observed effects of exercise in this study occur under the background of potential pathophysiological effects of isolation during postnatal neurodevelopment. Interestingly, with respect to the effects of isolation, two studies have observed a reduction on anxiety in individually housed males [41], [42].

### Reduced Anxiety-like Behaviors of Running Mice Are Not Related to Reduced Physiological Stress

Studies examining the effects of voluntary exercise on anxiety-related behavior have had contradicting results with respect to observed physiological changes related to stress. For example, while a group of studies did not observe changes in corticosterone levels [17], others have reported initial temporary increases in corticosterone levels in plasma, which eventually decreased to basal levels [43]. On the other hand, studies using forced swimming and restraint tests on their animal subjects reported increased corticosterone levels [44], [45], suggesting that the type of exercise and whether it is voluntary or forced may result in increased stress. Our results showed no significant difference between plasma corticosterone levels between exercised mice after four weeks of voluntary running, compared to sedentary controls, suggesting that the observed decrease in anxiety-like behavior in running animals observed by us is not related to alterations of corticosterone levels. This finding led us to propose that other types of molecular or biochemical changes in the brain might be associated to the anxiolytic effects of running.

### Voluntary Wheel Running Enhances Cortical Abundance of AA and DHA, while Reducing the Cortical Abundance of PA

Since polyunsaturated FA, such as DHA, have been shown to enhance the effects of running in the brain when used as dietary supplements [22], we set out to determine if voluntary running could induce changes in endogenously synthesized FA. Non-targeted lipidomics allowed examination of exercise-induced profiles of the total FA within the hippocampus, cortex and cerebellum. While the amygdala is an essential part of the limbic system that processes fear and anxiety [46], [47], we did not examine its neurolipidome in this study because exercise has been mostly associated to changes in the cerebellum [48]–[50], cortex [51]–[55], and hippocampus [56]–[61]. Hierarchical clustering analysis of the data obtained from the hippocampus, cerebellum, and cortex, revealed a clear pattern of separation with respect to the sedentary and running conditions of our study. Specifically, a separation with respect to FA profiles, particularly of reduction in PA abundance accompanied by an enhancement in AA and DHA abundances, was observed within the cortex, but not in the hippocampus or cerebellum of running animals compared to sedentary controls.

### Potential Significance of Cortical FA Modulation by Exercise

FA are important in membrane fluidity, cell-cell interactions, among other cellular functions. When freed from cellular membranes they also play important roles in intracellular signaling, intercellular communication, and cellular growth. AA and DHA are highly abundant in nervous tissue and are important molecules in neural development, functioning in ways that might extend into adulthood [23]–[25]. For example, AA and DHA are abundant FA in the growth cone and they both have been suggested to play important roles in the adult brain related to synaptic extension and dendritic arbor remodeling [23]–[25]. Such findings suggest that these FA might play a role in synaptic plasticity, learning, and memory processes. Indeed, dietary supplementation with DHA enhances learning and memory processes in rodents possibly by regulating cellular and molecular mechanisms associated to synaptic plasticity, transmission, and growth [21], [22]. Of interest to our findings is the fact that in such studies voluntary exercise had a synergistic effect with DHA dietary supplementation on learning and memory processes.

DHA seems to have an important role in cognition, anxiety-like behavior, and stress. Polyunsaturated FA supplementation by itself has been shown to enhance learning and memory in the Morris water maze [62]–[65], radial arm maze [66] and the Barnes maze [67]. Conversely, DHA dietary restriction impairs olfactory discrimination learning in rats [68], [69]. Interestingly, DHA has been found to lower anxiety-like behavior and stress in rodents. Studies in which rats were supplemented with linoleic or α linoleic acid and DHA, followed by EPM [70], [71] and light/dark box [63] tests, observed reduced anxiety-like behavior associated to the dietary supplementations. A separate study focusing on stress behavior, reported a reduction of stress in rats after DHA dietary supplementation [71]. Finally, the vast majority of clinical experimental findings suggest that DHA is involved in the modulation of anxiety-like behavior and stress responses since dietary supplementation with omega-3 FA, specifically DHA, has beneficial effects in the treatment of patients with depression and anxiety disorders [24], [72]–[74].

Inhibition of lipoxygenase and PLA_2_, which convert AA into various metabolites such as prostaglandins or leukotrienes, block calcium induced hippocampal dentate gyrus and CA1 long-term potentiation (LTP). These results provide evidence for the importance of AA in synaptic plasticity processes [75], [76]. Activation of N-Methyl-D-Aspartate (NMDA) receptors, in the presence of L-glutamate, induced the release of AA [77]. AA can also stimulate the activation of Protein Kinase C and the phosphorylation of GAP-43 [78], [79]. Moreover, administration of AA to aged rats results in faster learning and improved memory in the Morris Water Maze task [80]. Interestingly, AA metabolites are also precursors of the endogenous cannabinoid, anandamide [81], which also improves learning and memory [82]. More relevant to our discussion, anandamide has also been found to have an anxiolytic effects in rodents [83]. In addition, anandamide may provide a sense of well being, typically observed during voluntary exercise [19], [84], by activating the endocannabinoid systems and reward pathways in the brain [19], [84], [85]. In fact, endocannabinoids are released in the blood during exercise [86], [87].

PA is one of the most common saturated FA, being the end product in FA synthesis pathways. PA itself plays a significant role in a variety of cellular processes because it is an important substrate in the posttranslational modification process known as palmitoylation, in which it is used as a substrate for covalent modification of different proteins [88]. In the nervous system, protein palmitoylation enhances synaptic communication and cell signaling [89], [90]. Palmitoylation plays a key role in synapse stabilization by aiding the trafficking of PSD95 and glutamate receptors such as the α-amino-3-hydroxy-5-methyl-4-isoxazolepropionic acid receptor (AMPA-R), specifically palmitoylated in the transmembrane domain of the C terminus of its subunits, and the NMDA receptor, specifically palmitoylated on its NR2 subunit [91]–[93]. Thus, the observed reduction in cortical PA levels may be reflective of enhanced protein synthesis and subsequent palmitoylation activity, causing a reduction in the available stores of PA (as observed in our studies) and, more importantly, help target important neurotransmitter receptors into neuronal membranes.

In addition to its role in regulating protein function as a substrate for palmytoilation, PA can be intracellularly converted into other biologically active lipid signaling molecules. Of importance to our discussion is the fact that PA is converted in cells into palmitoylethanolamide (PEA) when combination with ethanolamide [94]. PEA acts as an endocannabinoid-like signaling molecule that does not bind plasma membrane cannabinoid receptors, but activates other targets of the endocannabinoid system including peroxisome proliferator-activated receptors [95]–[99]. Inhibition of FA Amide Hydrolase, the enzyme that cleaves PEA and endocannabinoid N-oleoyl ethanolamide (OEA), results in the elevation of and PEA/OEA levels decreasing anxiety-like behavior in rodents, as measured in the EPM [100]. Moreover, similar to exercise, PEA has been shown to have antidepressant effects [101]. Thus, the decrease in cortical PA reported here may be associated to its being used as a substrate for i) palmitoylation in order to enhance neurotransmission and cell signaling processes or ii) PEA synthesis, both being mechanisms that can help explain the anxiolytic effects of running observed by us in this study.

### Voluntary Wheel Running Enhances PLA_2_ Expression

Since our neurolipidomic studies demonstrated that voluntary running results in specific cortical increases of AA and DHA levels, we next set out to determine if such changes were associated with enhanced cortical PLA_2_ expression. The increase of total PLA_2_ associated to voluntary running, observed in our studies, provides a helpful clue for the reasons we observed increased AA and DHA levels in the cortex. The cortical running-associated increases in total PLA_2_ levels suggest that the specific cortical increases in AA and DHA observed in response to running, might be related to activation of PLA_2_ signaling. PLA_2_ is mostly present in the cytoplasm and in cytosolic vesicles [102]–[104]. Cytoplasmic PLA_2_ (cPLA_2_) is directly related to the modification of AA in the process of eicosanoid production, while Plasmalogen-Selective PLA_2_ (PlsEtn-PLA_2_) is the enzyme that hydrolyzes AA and DHA and releases these polyunsaturated FA from membranes into the cytoplasm [103]. In the case of cPLA_2_, when calcium levels increase in the cytoplasm, PLA_2_ is translocated into nuclear or other cellular membranes [105]. Once activated, its main function is to liberate phospholipids, such as AA and DHA, from membrane lipid bilayers. Finally, the possible synergistic effects resulting from enhanced AA and DHA release may also explain the anxiolytic effects of voluntary exercise that we observed in our studies and that support several other studies in the field.

In summary, our results provide further evidence supporting that young adult mice engaging in voluntary running, have decreased innate, non-learned anxiety-like behavior as measured in the EPM, an effect that results by potentially activating cortical signaling cascades involving or dependent on bioactive lipids, such as PA, AA, and DHA. Neurolipidomic analysis identified cortex specific differences in FA abundance in young adult running mice compared to their sedentary counterparts. Running animals showed cortex specific increases of AA and DHA levels coupled with cortex specific decreases of PA, compared to results obtained from sedentary controls. The running-induced cortical regulation of PA, AA, and DHA is possibly associated to the anxiolytic effects of exercise. The changes in the levels of these particular FA may provide individual or synergistic effects that may help explain the accumulating data concerning the benefits of exercise on mental health.

## Materials and Methods

### Animals

Young adult male mice (C57BL/6) of 8 to 9 weeks of age were obtained from a colony at the animal facilities of the University of Puerto Rico, Rio Piedras Campus. Prior to voluntary wheel running all mice were housed in groups with *ad libitum* access to food and water in a 12 h light-dark cycle. One day before the start of the running period, mice from each group were separated and placed individually for 24 h for acclimatization followed by individual housing with *ad libitum* access to food and water in a 12 h light-dark cycle during voluntary running period. All the procedures were approved by the Institutional Animal Care and Use Committee of the Río Piedras Campus of the University of Puerto Rico. As such, the procedures are in compliance with National Institutes of Health (NIH) guidelines for the care and use of laboratory animals (Department of Health and Human Services–NIH publication number 86–23).

### Voluntary Running Training

The animals were divided into two groups: the non-running animals (Sedentary) and the physically active animals (Running). Mice in the Sedentary group were housed individually in their corresponding home cages. Mice in the Running group were also housed individually, but their home cages were furnished with a running wheel. Animals in both groups had *ad libitum* access to food and water in a 12-hour light/dark cycle. Mice in the running group were placed them in their individual cages with a *Med Associates Wireless* running wheel for a period of a month. Mice in this group (Running) had continuous free access to the running wheel. Running data provided by the Med Associates program was collected for each animal through the running period. Data was collected and averaged per week (**Fig. 1**).

### EPM, Behavioral Postures, and RAB Measurements

The EPM paradigm has been extensively used to measure anxiety levels in rodents [12], [105]. The apparatus (Med Associates Inc, Vermont, USA) is in the shape of a cross with two open arms (10 cm width ×50 cm long) and two closed arms (10 cm width ×50 cm long) enclosed in two walls that are 30 cm high. All of the arms are 55 cm above the floor level. Immediately after a month of voluntary wheel running, mice were subjected to the EPM and their behavior was recorded using the Video maze software (Med Associates) for analysis. Experiments were conducted within an hour of the voluntary running period and at the end of their light cycle in a white room with a single light facing the EPM (Video Maze, 2008). Briefly, 15 sedentary and 16 running mice were divided into three sets in order to carry out EPM testing in three different sessions. Each EPM session examined a maximum of 10 to 11 animals per session (5 to 6 animals per group) and each session was done in different days. The task consisted of recording behaviors while on the EPM for a total of 5 min per mice. Every session started at 7:00 pm sharply and testing a complete set of mice lasted no longer than 1 hour and 20 minutes. Mice were carefully placed in the center square, the hub, facing the open arms. Several measures were taken: time spent in the open arms, time spent in the closed arms, entries to the open arms, entries to the closed arms, and time in the hub. In addition to the data provided by the EPM, the frequencies (number of times the mice performed the posture) of several behavioral postures were analyzed during the 5 min EPM test in accordance to previous studies [106]. Two observers blind to the group assignment of the animals, positioned themselves near each end of the covered arms of the maze to record four RABs. The following common RABs were observed: Stretch Attendance Posture (SAP: the animal stretches forward and then retracts to its original position), unprotected head dipping (UHD: head flexion below the edge of the open arms of the maze), and protected head dipping (PHD: head flexion from the covered arms or the hub towards the open arms edge). Rearing (the animal stands in its hind paws without support or against the walls of the closed arms) was analyzed as an exploratory behavior, and grooming was observed as avoidance behavior. These parameters were also employed to further assess anxiety related behaviors in the EPM [106].

### Blood collection

For measurement of corticosterone levels, either sedentary (N = 16) or running animals (N = 15) were first subjected to the EPM (as above) prior to blood collection. Immediately after each individual mouse ended its 5 minutes in the EPM, it was restrained by an experienced animal care technician and blood was collected through the eye using a fine crystal pipette tip according to the procedure approved by the Institutional Animal Care and Use Committee of the Río Piedras Campus of the University of Puerto Rico. This procedure lasted no more than 2 minutes per mice and collected between 200 μl to 400 μl of blood. After blood collection, plasma was obtained and stored in −80°C. Importantly, each of our three EPM sessions lasted up to 1 hour and 20 minutes (see above) and completion of blood collection per session lasted no longer than minutes 40. Therefore, both EPM testing and blood collection procedure lasted up to 2 hours for each of the three EPM and blood collection sessions. Thus, considering the temporal regulation and time restraints in the rhythm of corticosterone changes throughout the day, within-group time-dependent differences in corticosterone levels would be unlikely to occur in the short time window of 2 hours utilized by us to complete the testing and blood collection for all animals.

### Corticosterone extraction and enzyme immunoassay (EIA)

For this procedure we used the Enzo 96-well Corticosterone Enzyme Immunoassay kit (Enzo Life Science Inc. Farmingdale NY, USA.). Thawed plasma samples were used to extract corticosterone. The extractions started by adding 2.5 μl of the steroid displacement reagent (provided by the kit) to 97.5 μl of the plasma sample in a glass test tube. Next, 1 ml of ethyl acetate was added and mixed by hand shaking in a fume hood. The top clear ethyl acetate organic layer was carefully removed and placed in another clean test tube. This procedure was repeated two more times, the three organic layers were combined, and the ethyl acetate solvent was evaporated in a Savant. Dried extracted corticosterone was stored at −80°C in the glass test tube until used. The day of the EIA, the samples were re-suspended in 250 μl of Assay Buffer (provided by the kit), vortexed, and allowed to stand for 5 min. This last step was repeated twice. A final dilution of the extracted corticosterone samples was done by combining 1 μl of the concentrated sample with 25 μl of Assay Buffer. The EIA itself was read in a micro-plate reader (Termo Lab-systems Multi-scan RC) using a 405 nm filter. Absorbance was obtained for each sample and controls. A standard curve was generated using Average Absorbance versus Percent Bound. Known corticosterone concentrations were graphed using Logit-log paper. Correlations of the values of the samples compared to the standard curve were used to obtain an estimated amount of corticosterone (picograms of corticosterone/milliliters of plasma) in plasma samples from Running and Sedentary mice.

### Tissue preparation for FA Extraction

For each condition we used a total of 8 to 12 animals. Directly after voluntary wheel running, EPM testing, and blood collection, mice were killed by decapitation and brains were quickly removed and meticulously dissected in order to obtain the hippocampi, cortex and cerebella. Briefly, after extraction from the skull, the brains were rinsed in ice-cold 1% Phosphate Buffer Saline and set to harden on dry ice then. Next, we carefully separated the cerebellum, with a fine spatula, followed by the brain stem and cortex and the hippocampus. Hippocampi and cortices from two animals per condition were pooled together in order to obtain 100 mg of tissue for FA extraction. Extracted cerebelli from single animals per condition were used to obtain 100 mg of cerebellar tissue for FA analysis. Immediately after extraction, each tissue was placed in liquid nitrogen, weighed, and stored at −80^o^C.

### FA Extraction

Tissue samples were homogenized with 2 ml CHCl_3_/MeOH (1∶1) (Sigma-Aldrich Corp. St. Louis, MO, USA.) using a modification of the method of Bligh and Dyer [107]. The resulting CHCl_3_ phase was evaporated to dryness under vacuum and the samples were transmethylated with MeOH and HCl (Sigma-Aldrich Corp.) for 24 hours at −20^o^C. FA methyl esters were extracted in ether cleaned by filtering through silica gel column, evaporated, and dissolved in 5 ml of hexane. The profiling of FA methyl esters was performed using the Agilent 7890A series Gas Chromatograph (Agilent Technologies, Inc., Santa Clara, CA) equipped with a 30 m×0.25 mm special performance capillary column (HP-5MS) of polymethyl siloxane crosslinked with 5% phenyl methylpolysiloxane followed by mass spectrometry. Mass spectra were interpreted by comparison of retention times with a group of internal FA methyl ethers standards (Sigma) previously separated on the same GC using Agilent 5975C MS ChemStation software.

### Non-Targeted Neurolipidomics

Quantitation was carried out using each peak height area, sum of all peaks, and expressed as a ratio of total of all peaks. GC/MS detected 29 FA methyl ethers in the hippocampus; cortex and cerebellum of young adult (8 weeks) C57BL/6 male mice of both sedentary and running groups. There were significantly more peaks per sample such as FA-aldehydes, which were excluded from the analysis as they represented products of derivatization with MEOH/HCl. In addition, we excluded from the analysis the content of saturated FA: C15:0, C17:0, C19:0 and C23:0 (detected at very low level), which come directly from the diet and, thus, cannot be assessed in terms of FA metabolic changes induced by running due to the biosynthesis or conversion to monounsaturated FA by corresponding enzymes. Using GC/MS we searched for different FA identity peaks and abundances between groups and across brain regions. We focused on 16 FA (six saturated FA, five monounsaturated FA and five polyunsaturated FA) and determined their abundances. Percent of total for each individual FA was calculated by dividing the abundance peak of each FA by the summation of the abundances of all 16 FA, followed by multiplication of the resulting value by 100. Percents of total were subjected to bioinformatics analysis performed using web-based tools for quantitative metabolomics – Metaboanalyst [108].

### Protein extraction

To examine PLA_2_ protein levels after a month of voluntary wheel running, mice were anesthetized and decapitated immediately after training. Brains were obtained, chilled on ice cold PBS, and used to dissect a portion of the cortex area between −1.70 mm and −0.70 mm bregma points. Cortical tissue from 2 animals per condition were combined yielding one pool sample with a total of 6 pools (N = 6). Pooled tissue was weighed and stored at −80°C until used for protein extraction. Protein extracts were prepared as described by us previously [109]–[111]. In summary, tissues were homogenized in extraction buffer [30 mM HEPES/KOH, pH 7.9, 0.5 M KCl, 5 mM MgCl_2_, 1 mM EDTA, 2 mM dithiothreitol (DTT), 20% glycerol, 1 mM phenylmethylsulfonyl fluoride (PMSF), and 1 µg/ml each of leupeptin and aprotinin (Sigma-Aldrich Corp.)] and incubated for 1 h in ice. The extract was centrifuged at 14,000 rpm for 1 h at 4°C using centrifuge (Eppendorf 5415D, Hauppauge, NY, USA). The supernatant was then dialyzed for 5 h in dialysis buffer [30 mM HEPES/KOH, pH 7.9, 50 mM KCl, 2 mM EDTA, 5 mM MgCl_2_, 1 mM DTT, 10% glycerol, 1 mM PMSF, and 1 µg/ml each of leupeptin and aprotinin (Sigma-Aldrich Corp.)]. Dialyzed fractions with a Molecular Weight Cut Off (MWCO) of 2000 Da (Sigma-Aldrich Corp.) were centrifuged at 14,000 rpm for 30 min at 4°C. Protein extracts were stored at –80°C until used. The protein concentration was determined by the Bradford method as detailed by us previously [109]–[111].

### Western blotting

For Western Blotting, protein samples (20 μg) and 2 μl of the Odyssey Pre-stained Molecular Weight Marker (LI-COR Biosciences, Nebraska, USA) were first separated on a 10% sodium dodecyl sulfate-polyacrilamide gel electrophoresis (SDS-PAGE). The separated proteins in the gel were transferred to a nitrocellulose membrane (Sigma-Aldrich Corp.) using a semidry electroblotter system at 15V for 1 hour and 30 seconds (Fisher Scientific, Cayey, PR). Then, the membrane was blocked using a mixture of 5% non-fat milk and Oddysey Blocking Buffer (LI-COR Biosciences) overnight on an orbital shaker (Fisher Scientific). After 3 washes of 15 min each with PBS Tween-20 (PBS-T) (Sigma-Aldrich Corp.), the membrane was incubated with a mixture of two primary antibodies: 1∶1000 dilution of a rabbit polyclonal antibody raised against a synthetic non-phosphopeptide derived from human PLA_2_ (Abcam, Cambridge, MA) and 1∶10000 dilution of a mouse monoclonal antibody raised against β Actin (Sigma-Aldrich Corp.) at 4°C overnight. After 3 washes of 15 min, each with PBS Tween-20 (PBS-T), the membranes were incubated with a mixture of two secondary fluorescent antibodies: (1∶2000 dilution of donkey anti-rabbit IRDye680 and 1∶5000 dilution of donkey anti-mouse IRDye800) (LI-COR Biosciences) for 1 h at room temperature. The membrane was washed as previously mentioned and scanned and analyzed using the Odyssey Infrared Imaging System (LI-COR Biosciences). After background subtraction, intensities were normalized by dividing each PLA_2_ signals from the various samples analyzed by the corresponding β-Actin signal.

### Statistical Analysis

For all of the statistical analysis, except the Non-Targeted Neurolipidomics analysis, we used the Graph Pad Prism 5 statistical analysis software. After gathering the averaged distance the mice ran weekly we analyzed the data utilizing One Way Repeated Measures ANOVA followed by Bonferroni post-testing. For the analysis of the EPM behaviors depicted in **Fig. 2A–D**, which included the preference index, time spent in the areas of the maze, explorations, and arm entries, Two-Way ANOVAs followed by Bonferroni post-hoc tests were used. The data on RABs and exploratory behaviors assessed in the EPM and depicted in **Fig. 2E**, was analyzed with a General Linear Model – MANOVA using the StatPlus®:mac software. Moreover, to analyze corticosterone concentrations and PLA_2_ protein immunodetection we used an Unpaired Student's T test. Lipid abundance analysis was obtained by using a Two-Way ANOVA statistical measures accompanied a multiple testing using Bonferroni. For Non-Targeted Neurolipidomics analysis, we used the Metaboanalyst web server, which aided in metabolomic data analysis and interpretation. Briefly, samples were normalized by column-wise normalization using autoscaling transformation (i.e. mean-centered and divided by the standard deviation of each variable). The dendrogram and heatmap were produced by applying Euclidean dissimilarities measures and the Ward clustering algorithm to show the hierachical clustering patterns among the samples. Finally, the intrinsic variations within FA data sets in each brain region of sedentary and running mice were examined by Principal Component Analysis (PCA). Principal component 1 (PC1) and principal component 2 (PC2) were used to discriminate differences between groups.

## Acknowledgments

We thank Carlos I. Rodríguez for his technical assistance in the behavioral studies, Luis Rosario, and Andrés Rodríguez for their technical assistance in the Animal House Facility of the University of Puerto Rico, Río Piedras.
